# Early persistence on therapy impacts drug-free remission: a case-control study in a cohort of Hispanic patients with recent-onset rheumatoid arthritis

**DOI:** 10.1186/s13075-022-02884-w

**Published:** 2022-08-12

**Authors:** Irazú Contreras-Yáñez, Guillermo Arturo Guaracha-Basáñez, Maximiliano Cuevas-Montoya, José de Jesús Hernández-Bautista, Virginia Pascual-Ramos

**Affiliations:** 1 Department of Immunology and Rheumatology, Instituto Nacional de Ciencias Médicas y Nutrición Salvador-Zubirán (INCMyN-SZ), Vasco de Quiroga 15, colonia Belisario Domínguez Sección XVI, Tlalpan, 14080 Mexico City, Mexico; 2 Emergency Medicine Department, Instituto Nacional de Ciencias Médicas y Nutrición Salvador-Zubirán (INCMyN-SZ), Vasco de Quiroga 15, colonia Belisario Domínguez Sección XVI, Tlalpan, 14080 Mexico City, Mexico

**Keywords:** Persistence, Drug-free remission, Rheumatoid arthritis

## Abstract

**Background:**

Medication adherence is suboptimal in rheumatoid arthritis (RA) patients and impacts outcomes. DMARD-free remission (DFR) is a sustainable and achievable outcome in a minority of RA patients. Different factors have been associated with DFR, although persistence in therapy (PT), a component of the adherence construct, has never been examined. The study’s primary aim was to investigate the impact of PT’s characteristics on DFR in a cohort of Hispanic patients with recent-onset RA.

**Methods:**

A single data abstractor reviewed the charts from 209 early (symptoms duration ≤ 1 year) RA patients. All the patients had prospective assessments of disease activity and PT and at least 1 year of follow-up, which was required for the DFR definition. DFR was defined when patients achieved ≥ 1 year of continuous Disease Activity Score-28 joints evaluated ≤ 2.6, without DMARDs and corticosteroids. PT was defined based on pre-specified criteria and recorded through an interview from 2004 to 2008 and thereafter through a questionnaire. Cases (patients who achieved ≥ 1 DFR status) were paired with controls (patients who never achieved DFR during their entire follow-up) according to ten relevant variables (1:2). Cox regression analysis estimated hazard ratios (HRs) for DFR according to two characteristics of PT: the % of the patient follow-up PT and early PT (first 2 years of patients’ follow-up).

**Results:**

In March 2022, the population had 112 (55–181) patient/years follow-up. There were 23 patients (11%) with DFR after 74 months (44–122) of follow-up, and the DFR status was maintained during 48 months (18–82). Early PT was associated with DFR, while the % of the patient follow-up PT was not: HR = 3.84 [1.13–13.07] when the model was adjusted for cumulative *N* of DMARDs/patient and 3.16 [1.14–8.77] when also adjusted for baseline SF-36 physical component score. A lower *N* of cumulative DMARDs/patient was also retained in the models. Receiving operating curve to define the best cutoff of patient follow-up being PT to predict DFR was 21 months: sensitivity of 0.739, specificity of 0.717, and area under the curve of 0.682 (0.544–0.821).

**Conclusions:**

DFR status might be added to the benefits of adhering to prescribed treatment.

**Supplementary Information:**

The online version contains supplementary material available at 10.1186/s13075-022-02884-w.

## Background

Current treatment guidelines for rheumatoid arthritis (RA) suggest tapering disease-modifying anti-rheumatic drugs (DMARDs) when patients are in sustained remission (SR) [1]. Two recent systematic literature and narrative reviews inform that with a treat-to-target approach, an increasing number of patients might achieve and sustain DMARD-free remission (DFR), with frequencies ranging from 3.6 to 22% of the patients [2, 3]. (Sustained) DFR has nowadays considered the closest state to RA cure [4] and, from the patient perspective, has been associated with the reversal of functional disability and the resolution of symptoms such as fatigue [5]. Several factors had been demonstrated to predict the successful maintenance of remission after classical DMARDs withdrawal. The absence of disease-specific autoantibodies is the most consistent and best predictor [2, 3, 6]. Meanwhile, symptom duration has arisen conflicting results, which might be explained by a non-linear association with DFR but refined to a short period, the window of opportunity [2, 3, 7]. Additional factors identified are a longer duration of SR before the drug withdrawal [8], lower disease activity at the time of treatment cessation [9–12], using methotrexate as the last DMARD before withdrawal [9], circulating inflammatory biomarkers and peripheral CD4+ T-cell gene expression [11], and a model that combined RA quality of life (QoL) score, musculoskeletal ultrasound-derived information, and the percentage of inflammation-related T-cell [13].

Tapering DMARDs, notably methotrexate, is desirable for RA patients concerned about long-term side effects and the burden of taking tablets or self-injecting if they are well [14]. These impact patient adherence to the prescribed treatment, which reflects the extent to which patients take their medication as prescribed [15, 16]. Previous literature reviews highlight that among patients with RA, adherence to DMARDs is suboptimal, and poor adherence affects 20 to 70% of the patients during their follow-up [17–21]. Inadequate medication adherence includes three major components (persistence, initiation adherence, and execution adherence) and causes a negative impact on the different patient and physician-reported outcomes, which our group has confirmed in Hispanic RA patients [17–25]. Meanwhile, the impact of medication adherence and its components on DFR, a realistic and achievable goal, has not been previously examined.

The study aimed to investigate the impact of persistence on therapy (PT)’s characteristics on DFR in an inception and ongoing cohort of Hispanic patients with recent-onset RA. We additionally described the DFR phenomenon.

## Methods

### Setting and study population

Patients with RA were identified from the early arthritis clinic of a national referral center for rheumatic diseases located in Mexico City, the Instituto Nacional de Ciencias Médicas y Nutrición Salvador Zubirán. Patients entering the clinic had been previously described [23–25]. They had a disease duration of less than a year when first evaluated and were assessed every 2 months during the first 2 years of follow-up. After that, visits were scheduled every 2, 4, or 6 months, depending on the patients and disease characteristics.

Treatment was prescribed by the rheumatologist in charge of the clinic, who embraced paradigmatic changes in treating the disease over the years [26, 27]. During the first years, she/he adopted aggressive and tight control of inflammation, considering the critical nature of the early disease and the functional deterioration due to treatment delay [26, 28]. A decade later, this concept was refined and formulated as the “treat-to-target” (T2T)-oriented approach [27] and was incorporated into patient management.

At study entry, the primary rheumatologist recorded a complete medical history, demographic data, and disease-specific auto-antibodies (rheumatoid factor [RF] and antibodies to citrullinated proteins [ACPA]). Standardized follow-up evaluations included extended joint counts, patient (PROs)- and physician-reported outcomes, comorbidity, treatment assessment, laboratory parameters, and hand and feet X-rays (on an annual basis) [23–25].

From the beginning of the clinic (2004), the primary rheumatologist prospectively assessed the patient’s medication behavior in standardized formats. From 2004 to 2008, she/he evaluated persistence through an interview conducted at every visit. Patients reported the names, doses, and schedules of DMARDs and corticosteroids they had taken since the previous visit. Then, patients were asked about missing and incorrect medications, quantities, and schedules. The rheumatologist compared the last prescription and the actual treatment and recorded the number of days of missing drugs. From 2008 onwards, persistence in therapy was assessed through the compliance questionnaire, with good sensitivity and satisfactory specificity to detect persistence [23].

### Study design

A nested within a cohort case-control study was designed to accomplish the primary objective. Cases were defined as patients who achieved at least one DFR status and controls as patients who never achieved DFR during their entire follow-up. Controls were paired to cases (2:1) according to sex, age at RA diagnosis (± 15 years), education, socioeconomic status, presence of RF and APCA at baseline, baseline Disease Activity Score-28 joints evaluated (DAS28) European League Against Rheumatism (EULAR) category [29], corticosteroid use during the first year of follow-up, number of DMARDs/patient during the first year, and baseline erosions.

### Data collection

Initiating in January 2022 and ending in March 2022, a single and trained data abstractor retrospectively reviewed all the charts and corroborated the integrity of the data collected. An independent observer confirmed all the DFR periods and PT statuses.

### Definitions

*DFR status* was defined when patients achieved SR (at least 1 year of continuous follow-up clinical assessments with DAS28 ≤ 2.6) without concomitant DMARDs and corticosteroids (any route). The definition combined characteristics from previously published definitions [2, 3].

According to the interview, *non-PT* was defined as the omission for ≥ 7 consecutive days of at least one DMARD and corticosteroids. Regarding methotrexate, at least one missing weekly dose was considered non-persistence. According to the compliance questionnaire [23], a patient was considered to be non-PT if in item 10 (“In the past 6 months, how often do you completely stop taking your DMARDs?”), boxes 2 (“sometimes”), 3 (“almost always”), and 4 (“always”) were filled.

### Statistical analysis

Descriptive statistics were used. The *χ*^2^ and Mann-Whitney *U* tests were used to compare the non-normally distributed variables according to their category.

For each patient, PT was calculated as the percentage of the entire patient follow-up (up to the first DFR status for cases or equivalent for controls) that he/she was non-PT-free. Also, early PT was defined if continuous PT during the first 2 years of follow-up was identified.

Cox proportional hazard regression analysis was used to estimate the hazard ratios (HRs) and their respective 95% confidence interval (CI) for DFR according to PT’s characteristics. Two PT characteristics were included: % of the patient follow-up PT and/or early PT. We initially performed a univariate analysis and then performed a multivariate analysis to adjust for potential confounders (a *p* cutoff ≤ 0.05 in the univariate analysis was considered to include the variables): model 1 was adjusted for baseline 36-Item Short Form Survey (SF-36) physical component score, model 2 was adjusted for the cumulative number of DMARDs/patient during the first 2 years of follow-up, and model 3 included early PT and was adjusted for baseline SF-36 physical component score and number of DMARDs/patient. We also switched in the models the % of the patient’s follow-up PT to the patient achievement of PT ≥ 80% of his/her follow-up.

Receiving operating curve (ROC) was used to define the best cutoff for continuous patient follow-up on PT to achieve (the first) DFR status.

All analyses were performed using SPSS (version 21.0, IBM Corp., Armonk, NY, USA).

### Ethics

The study was approved by the Intutional Review Board (Comités de Ética e Investigación del Instituto Nacional de Ciencias Médicas y Nutrición Salvador Zubirán). All the patients provided written informed consent for clinical follow-ups when entering the clinic. They provided additional written permission to review each patient’s chart and present data in scientific publications.

## Results

### Study population characteristics

**Fig. 1:**
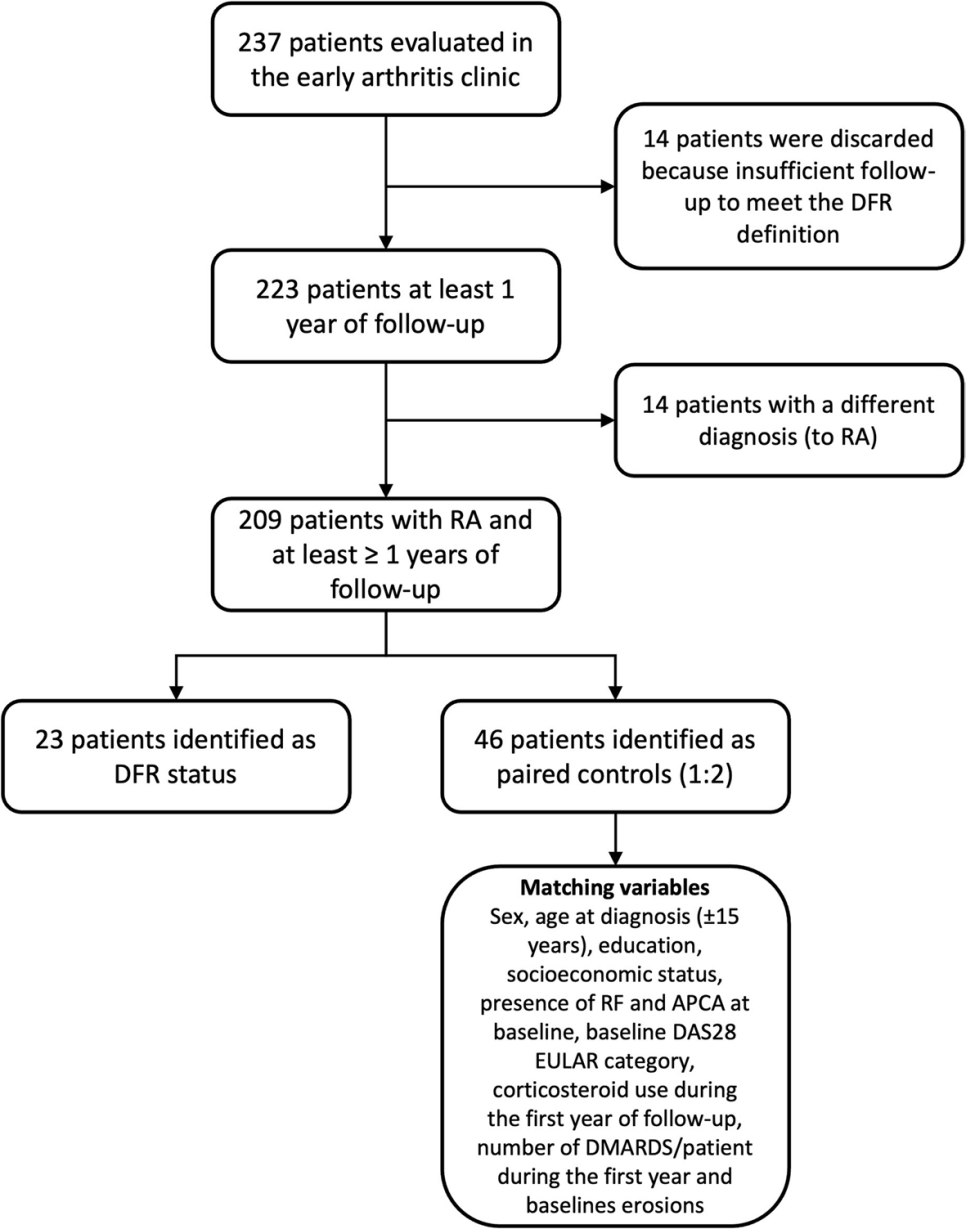
Patient’s flowchart

Up to March 2022, 237 patients had been evaluated in the early arthritis clinic, and 223 had at least 1 year of follow-up, which was required as per the DFR definition. Fourteen patients had a different diagnosis (to recent-onset RA) and were eliminated from the analysis (Fig. 1).

The baseline patient characteristics of the remaining 209 patients are summarized in Table 1. Briefly, they were primarily middle-aged women with medium-low socioeconomic status. The majority of them had RF and ACPA, while a few had baseline erosions. Patients had significant clinical and serological disease activity, translating into disability and poor quality of life (QoL). In addition, all the patients have indicated DMARDs, and almost half of them have corticosteroids.

**Table 1:** Population characteristics at baseline and comparison between cases (patients with DFR status) and their paired controls

	Study population, ***n*** = 209	Cases, ***n*** = 23	Controls, ***N*** = 46	***p***
**Sociodemographic characteristics**				
Years of age^a^	38.3 (27.2–48.3)	39 (25.5–44.2)	36.3 (27.9–46.6)	0.656
Female sex^b^	183 (87.6)	18 (78.3)	36 (78.3)	1
Years of formal education^a^	12 (9–15)	12 (9–15)	11.5 (9–14)	0.5
Medium-low SE status^b^	188 (90)	20 (87)	39 (84.8)	1
**RA-related characteristics**				
Months of disease duration^a^	5.1 (3–6.8)	5.2 (2.5–7)	4.9 (2.9–7.1)	0.775
RF (positive titers)^b^	173 (82.8)	13 (56.5)	33 (71.7)	0.280
ACPA (positive titers)^b^	172 (82.3)	11 (47.8)	28 (60.9)	0.318
Erosions^b^	21 (10.1)	1 (4.3)	5 (10.9)	0.656
DAS28^a^	5.8 (4.6–6.8)	5.3 (4.5–6.4)	5.6 (4.2–6.5)	0.990
Swollen joint count^a^	13 (8–18)	10 (6–16)	11.5 (7.8–18)	0.389
Tender joint count^a^	13 (7–18)	12 (6–18)	12 (7–16)	0.784
ESR, mm/H^a^	21 (10–39)	18 (13–31)	19 (7.5–35.3)	0.731
CRP, mg/dL^a^	0.7 (0.2–2.5)	0.3 (0.1–1.5)	0.6 (0.2–2.4)	0.074
**Patient-reported outcomes measures**				
HAQ score (0–3)^a^	1.4 (0.9–2)	1.3 (0.8–1.8)	1.1 (0.4–1.8)	0.342
SF36 physical-component score (0–100)^a^	35 (23.5–54.2)	30.7 (22.3–51.3)	44.7 (27.3–66.6)	0.044
SF36 mental-component score (0–100)^a^	44.6 (29.7–59.4)	39.5 (28.8–66)	51.7 (37.3–68)	0.208
Patient-overall disease-VAS^a^	51 (28.5–74)	44 (18.5–68)	44.5 (23.8–73.8)	0.593
**RA-related treatment**				
With corticosteroids^b^	109 (52.2)	9 (39.1)	18 (39.1)	1
With DMARDs^b^	209 (100)	23 (100)	46 (100)	NA
DMARDs/patient	2 (1–2)	2 (1–2)	2 (1–2)	0.265

*SE* socio-economic, *RF* rheumatoid factor, *ACPA* antibodies to citrullinated proteins, *DAS28* Disease Activity Score (28 joints), *ESR* erythrocyte sedimentation rate, *CRP* C-reactive protein, *VAS* visual analog scale, *HAQ* Health Assessment Questionnaire, *SF-36* Short Form 36 Items, *DMARDs* disease-modifying anti-rheumatic drugs

^a^Median (IQR)

^b^Number (%) of patients

### Description of DFR phenomenon

Up to March 2022, the early arthritis clinic had 112 (55–181) patient/years follow-up. There were 23 patients (11%) who achieved DFR status (cases) after 74 months (44–122) of follow-up; in them, DFR status was maintained during 48 months (18–82). Their DAS28 at DFR was 1.7 (1–2.1), and 20 of them (87%) additionally had Boolean remission and according to the Simplified Disease Activity Index (SDAI) [30].

**Fig. 2:**
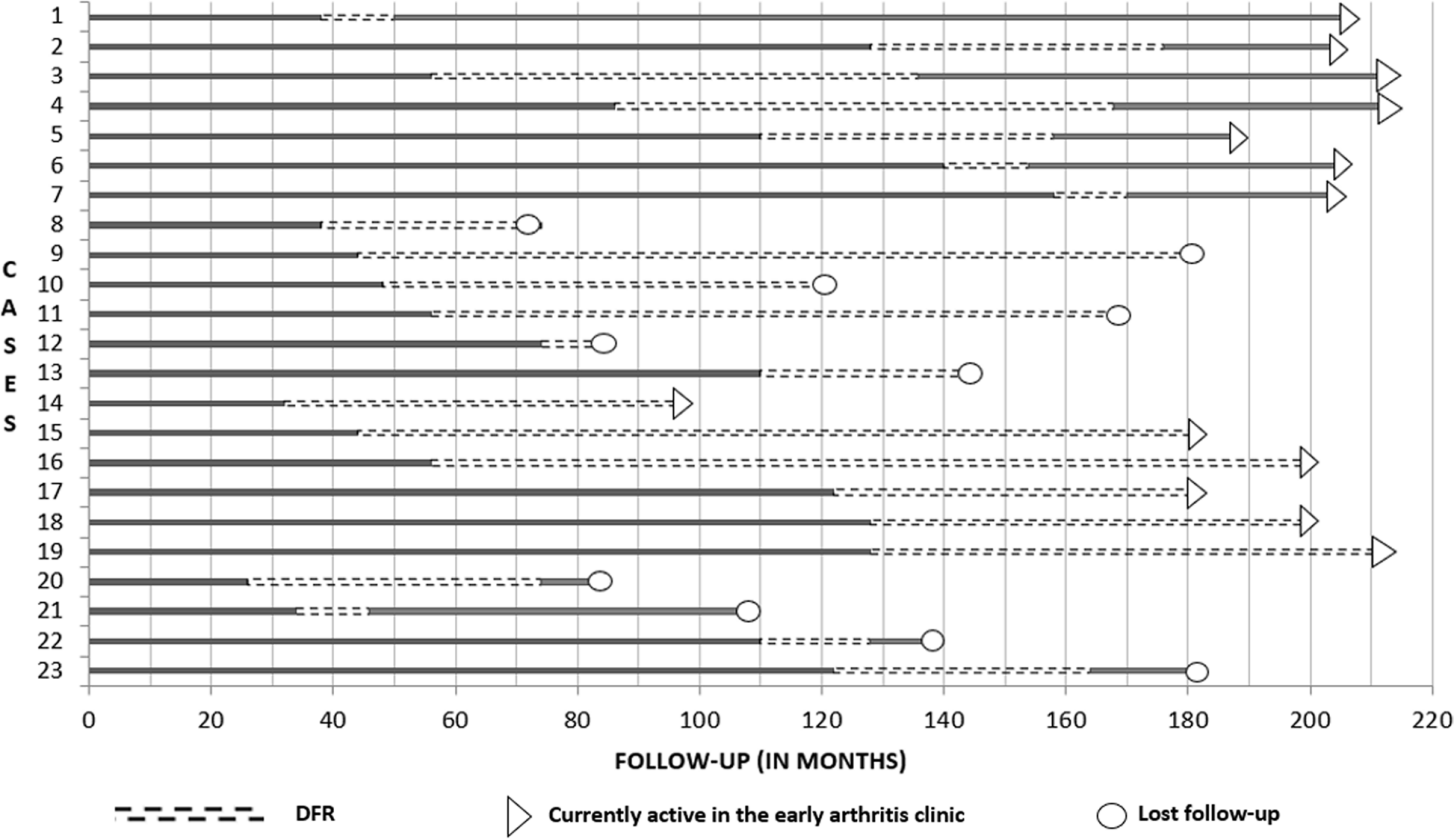
DFR behavior of the 23 cases

Figure 2 summarizes the DFR behavior of the 23 cases. Six cases (26.1%) presented health care drop-out while in DFR, and six additional patients (26.1%) are currently active in the early arthritis clinic and maintain their DFR status. The remaining 11 patients (47.8%) lost DFR status; four patients had health care drop-outs, while seven are currently active in the early arthritis clinic. We further compared baseline characteristics between patients who lost DFR status and their counterparts, and no significant differences were identified (Additional file 1: Table S1).

### Comparison of cases and controls

The 23 cases were paired with 46 controls. Additional file  2: Table S2 summarizes the matching criteria, and the percentage of case-controls matching achieved.

Cases and controls were similar in baseline characteristics, but the SF36 physical component score was lower among the cases (Table 1).

Table 2 summarizes the comparison of cumulative (up to DFR status for cases or equivalent for controls) DAS28 joints evaluated [31], the number of DMARDs/patient, and PT characteristics between cases and controls. As observed, cases received a lower number of DMARDs/patient and were more frequently early PT when compared to controls; also, cases tend to be more frequently PT than controls.

**Table 2:** Comparison of cumulative disease activity, treatment, and PT behavior between cases and controls

	Cases, ***N*** = 23	Controls, ***N*** = 46	***p***
**Disease activity**			
DAS28	2.2 (1.9–2.6)	2.4 (1.6–2.9)	0.401
**Treatment**			
Number of DMARDs/patient	1.8 (1.2–2)	2.3 (1.6–2.8)	0.003
**PT characteristics**			
% of follow-up with PT	92 (58–100)	67 (47–88)	0.123
Early PT^a^	17 (73.9)	17 (37)	0.005
PT ≥ 80% of follow-up^a^	14 (60.9)	17 (37)	0.075

Data presented as median (IQR) and ^a^number (%) of patients

### Impact of PT on DFR phenomenon

Table 3 summarizes the results from Cox proportional hazard regression analysis. In models 2 and 3, early PT (HR 3.84 [1.13–13.07] and 3.16 [1.14–8.77], respectively) and a lower number of DMARDs/patient during the first 2 years of follow-up (HR 0.19 [0.08–0.45] and 0.12 [0.07–0.41], respectively) were associated with DFR status.

**Table 3:** Risk of DFR according to PT’s characteristics

	Model 1	Model 2	Model 3
	HR (95% CI), *p* value		
% of the patient follow-up PT	1.00 (0.98–1.02), 0.659	1.00 (0.98–1.02), 0.959	
Early PT	1.79 (0.59–5.46), 0.303	3.84 (1.13–13.07), 0.031	3.16 (1.14–8.77), 0.027
Baseline SF-36 physical component score	0.99 (0.96–1.02), 0.47		0.98 (0.96–1.01), 0.136
Cumulative (up to the first 2 years of follow-up) number of DMARDs/patient		0.19 (0.08–0.45), ≤ 0.001	0.12 (0.07–0.41), ≤ 0.001

*HR* hazard ratio, *CI* confidence interval, *PT* persistence on therapy, *SF-36* Short Form 36, *DMARDs* disease-modifying anti-rheumatic drugs

**Fig. 3:**
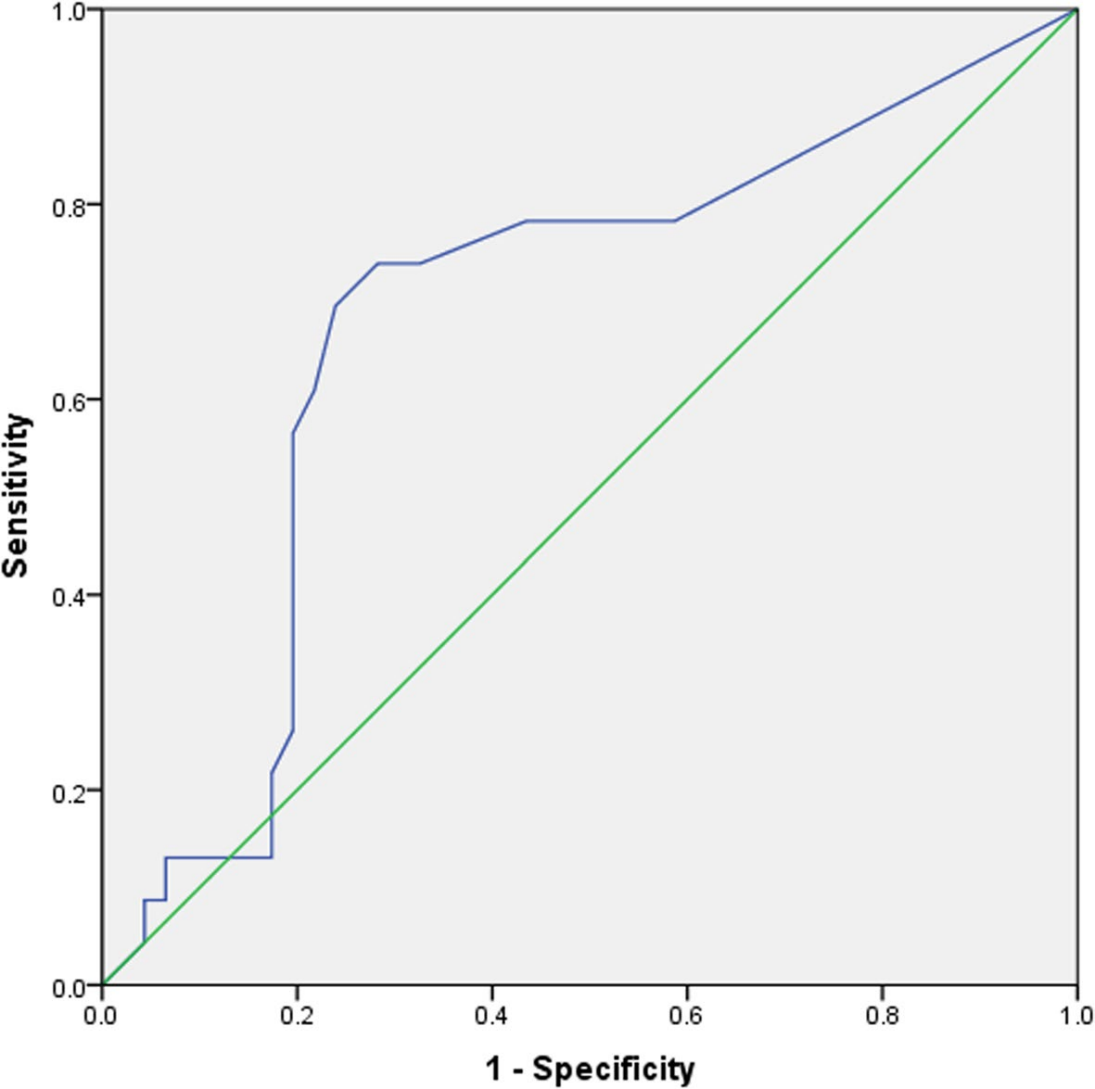
ROC for patient’s follow-up PT cutoff to predict DFR

Finally, ROC to define the best cutoff of patient follow-up being PT to predict DFR was 21 months: sensitivity of 0.739, specificity of 0.717, and AUC of 0.682 (0.544–0.821), as shown in Fig. 3.

## Discussion

Hispanic RA patients present distinctive characteristics such as a lower age at presentation, an extreme female preponderance, and a less severe disease expression; however, they are frequently uninsured with low socioeconomic status and lesser educated than patients from developed countries [32, 33]. These are known variables to impact patients’ commitment to the prescribed treatment. Published observations have shown that poor adherence to therapy is a generalized phenomenon among RA patients, potentially reversible, and associated with a wide variety of unfavorable outcomes [15]. Most relevant include increased disease activity, disease flares, and health care drop-outs; worse PROs (such as increased pain, disability, and reduced QoL); and decreased rates of remission [16–25, 34–40]. In contrast, PT translates into long-standing significant improvements, which additionally appear early on [24].

The present study involved a well-characterized inception ongoing cohort of Hispanic patients with early RA. Patients had a long-term standardized follow-up, which included the prospective assessment of PT (a component of the adherence construct), disease activity-related outcomes, and current treatment. Traditional DMARDs given according to a T2T strategy were the mainstay of treatment. In addition, we followed cohort methodological recommendations to ensure the quality of the data [41]. Accordingly, we consider the results to reflect patients’ daily conditions and are of practical relevance.

The study complements the current knowledge on the topic and extends the benefits of the patients’ adherence to DMARDs to DFR status. In particular, early PT, which was defined as occurring during the first 2 years of patients’ cohort enrollment, was associated with DFR, while the percentage of the patient’s follow-up PT was not. Interestingly, 21 months was the best cutoff of the patient’s follow-up PT to predict DFR. The results are in accordance with the window of opportunity concept that states there are superior clinical responses and the potential for remission (we might extend to DFR) when patients with RA are managed early and aggressively with DMARDs [42–45]. Also, the 21-month cutoff highlights that patients need to adhere (closely and) early on during their follow-up to treatment recommendations if significant outcomes are desired. In regard to the predictors of DFR, different factors have been described, although PT has never been examined [2, 3, 6, 12, 45, 46]. Genetical [2], immunological [2, 3], imaging [3], and clinical associations [3] with predictors of DFR have been consistently identified, including the presence of autoantibodies, absence of power Doppler signal on musculoskeletal ultrasound, lower disease activity, and PROs at treatment cessation. Our study design is unique in addressing such a hypothesis, while we could not confirm additional factors. Moreover, some authors argued that the constant frequency of DFR in different cohorts of patients over time suggests it most likely represents spontaneous remission without any direct relationship to treatment [6]. However, adherence constructs had been related to different outcomes, including SR, which highlights results plausibility [43].

A lower *N* of DMARDs/patient during the first 2 years of follow-up was retained in the model. It could be explained by patients with DFR perceived by the treating rheumatologist with lower disease activity at baseline and might be indicated a lesser intensive treatment. There are circumstances where clinicians disagree with the disease activity assessment from a composite index and, therefore, would prefer to make a treatment decision based on their clinical judgment [47]. Also, patients were treated with a T2T approach, a complex process involving aggressive early management with several therapy modifications requiring frequent close monitoring of disease activity and drug toxicities, and more liable to suboptimal adherence in real-life clinical practice [47]. More complex therapeutic regimens have been associated with lower compliance, confirmed in our population [24].

Finally, we observed that DFR was achieved by 11% of the patients after 74 months of follow-up; also, DFR was sustained for 48 months, while almost half of the patients flared subsequently. Our prevalence figure for DFR status follows literature reviews and overviews that include data from early arthritis cohorts [2, 3, 6, 12, 45, 46]. Also, substantial DFR durations [9, 48] and a similar percentage of flares [45, 49, 50] had been previously described in patients who achieved DFR status. Finally, we did not identify baseline differences between patients who maintained DFR and their counterparts, which was probably related to the limited number of patients in either group.

Some limitations need to be addressed. First, the study was performed in a RA population with distinct characteristics, limiting the results’ generalization. Second, the power of the study was limited by the occurrence of only 23 cases which allows 2–3 potential predictors per model. Third, we did not use a single validated questionnaire to assess PT and arbitrarily used a lag time of 1 week to define therapy discontinuation; nonetheless, our rate of non-PT was consistent with similar measures in related studies [25, 35, 51, 52]. Fourth, we did not consider physician adherence to the T2T strategy, associated with better outcomes, such as higher remission rates and improved disease activity. Lastly, DFR status was defined based on sustained DAS28 cutoff, which might not reflect a real (sustained) remission status.

## Conclusions

In summary, DFR is the most desirable outcome for RA patients and a proxy for disease cure. It can be achieved in a real-world setting, in a significant number of Hispanic patients with the recent-onset disease, and treated with a T2T approach. The current study extends the benefits of adhering to the prescribed treatment to DFR status and highlights that persistence with prescribed therapy during the first 21 months of disease follow-up might be associated with the most relevant outcome.

## Supplementary Information

**Additional file 1: Table S1.** Baseline population characteristics between patients who maintained DFR status and their counterparts.

**Additional file 2: Table S2.** Matching criteria and percentage achieved in the controls’ selection.

## Data Availability

All data that support our findings are contained within the manuscript. Requests for further details on the dataset and queries related to data sharing arrangements may be submitted to the corresponding author.

## Abbreviations

RA: Rheumatoid arthritis
DMARDs: Disease-modifying anti-rheumatic drugs
SR: Sustained remission
DFR: DMARD-free remission
QoL: Quality of life
PT: Persistence in therapy
T2T: Treat-to-target
RF: Rheumatoid factor
ACPA: Antibodies to citrullinated proteins
PROs: Patient-reported outcomes
DAS28: Disease Activity Score-28 joints evaluated
EULAR: European League Against Rheumatism
HRs: Hazard ratios
CI: Confidence interval
SF-36: 36-Item Short Form Survey
ROC: Receiving operating curve
SDAI: Simplified Disease Activity Index
