# Supplementary material for: Early persistence on therapy impacts drug-free remission: a case-control study in a cohort of Hispanic patients with recent-onset rheumatoid arthritis
Source: Arthritis Res Ther. 2022 Aug 12;24:193. doi: 10.1186/s13075-022-02884-w (PMC9373313; doi:10.1186/s13075-022-02884-w)
Supplement: Supplementary file 1 — Additional file 1: Table S1. Baseline population characteristics between patients who maintained DFR status and their counterparts. [file 13075_2022_2884_MOESM1_ESM.pdf]

**Supplementary Table 1. Baseline population characteristics between patients who maintained DFR status and their counterparts.**

| | Patients who lost DFR, n=11 | Patients who maintained DFR, n=12 | p |
|---|---|---|---|
| **Sociodemographic characteristics** | | | |
| Years of age* | 27.2 (19.3-44.2) | 39.7 (28.4-49.7) | 0.196 |
| Female sex† | 9 (81.8) | 9 (75) | 1 |
| Years of formal education* | 12 (9-13) | 11.5 (9-16) | 0.685 |
| Medium-low SE status† | 10 (90.9) | 10 (83.3) | 1 |
| **RA-related characteristics** | | | |
| Months of disease duration * | 4.2 (1.4-6.8) | 6.1 (3.7-8) | 0.116 |
| RF (positive titers)† | 8 (72.7) | 5 (41.7) | 0.214 |
| ACPA (positive titers) † | 6 (54.5) | 5 (41.7) | 0.684 |
| Erosions† | 1 (9.1) | 0 | 0.478 |
| DAS28* | 5.1 (4.6-6.5) | 5.3 (4.2-6.2) | 0.758 |
| Swollen joint count* | 8 (6-16) | 10 (5.3-17) | 0.877 |
| Tender joint count* | 12 (7-18) | 11 (5-17.5) | 0.758 |
| ESR, mm/H* | 17 (14-34) | 19 (12.3-28.8) | 0.805 |
| CRP, mg/dL* | 0.3 (0.1-0.7) | 0.4 (0.1-1.9) | 0.441 |
| **Patient-reported outcomes measures** | | | |
| HAQ score (0-3)* | 1.4 (1-1.6) | 1.1 (0.5-2.1) | 0.853 |
| SF36 physical-component score (0-100)* | 30.7 (22.2-50.3) | 35.7 (23.9-53.8) | 0.712 |
| SF36 mental-component score (0-100)* | 63.2 (31.3-76.4) | 35.4 (21.6-53.2) | 0.157 |
| Patient-overall disease-VAS* | 47 (28-68) | 42 (10.3-64.8) | 0.689 |
| **RA-related treatment** | | | |
| With corticosteroids † | 4 (36.4) | 5 (41.7) | 1 |
| With DMARDs † | 11 (100) | 12 (100) | NA |
| DMARDs/patient | 2 (1-3) | 2 (1.3-2) | 0.581 |

**Median (IQR). †Number (%) of patients. SE=Socio-economic. RF=Rheumathoid factor. ACPA=Antibodies to citrullinated proteins. DAS28=Disease activity score (28 joints). ESR=Erythrocyte sedimentation rate. CRP=C reactive protein. Vas=Visual analog scale. HAQ=Health assessment questionnaire. SF-36=Short form 36 items. DMARDs=Disease modifying anti-rheumatic drugs.*
