# Supplementary material for: Early persistence on therapy impacts drug-free remission: a case-control study in a cohort of Hispanic patients with recent-onset rheumatoid arthritis
Source: Arthritis Res Ther. 2022 Aug 12;24:193. doi: 10.1186/s13075-022-02884-w (PMC9373313; doi:10.1186/s13075-022-02884-w)
Supplement: Supplementary file 2 — Additional file 2: Table S2. Matching criteria and percentage achieved in the controls’ selection. [file 13075_2022_2884_MOESM2_ESM.pdf]

Supplementary Table. Matching criteria and percentage achieved in the controls' selection.

Matching criteria	%
Sex	100
Age at RA diagnosis (±15 years)	100
Education level (up to middle-high/>middle-high education)	100
Socioeconomic status (medium-low/above)	97.8
Presence of RF	84.8
Presence of ACPA	76.9
DAS28 EULAR category	89
First-year corticosteroid use	100
First-year cumulative number of DMARDs/patient	90
Baseline erosions	93.5
